# Cleavage of the *moaX*-encoded fused molybdopterin synthase from *Mycobacterium tuberculosis* is necessary for activity

**DOI:** 10.1186/s12866-015-0355-2

**Published:** 2015-02-06

**Authors:** Nicole C Narrandes, Edith Erika Machowski, Valerie Mizrahi, Bavesh D Kana

**Affiliations:** DST/NRF Centre of Excellence for Biomedical TB Research, Faculty of Health Sciences, University of the Witwatersrand, National Health Laboratory Service, P.O. Box 1038, Johannesburg, 2000 South Africa; MRC/NHLS/UCT Molecular Mycobacteriology Research Unit and DST/NRF Centre of Excellence for Biomedical TB Research, Institute of Infectious Disease & Molecular Medicine and Division of Medical Microbiology, University of Cape Town, Cape Town, South Africa

**Keywords:** MoCo biosynthesis, Molybdopterin synthase, mycobacteria, MoaX, MoaD, MoaE, Nitrate reductase

## Abstract

**Background:**

Molybdopterin cofactor (MoCo) biosynthesis in *Mycobacterium tuberculosis* is associated with a multiplicity of genes encoding several enzymes in the pathway, including the molybdopterin (MPT) synthase, a hetero tetramer comprising two MoaD and two MoaE subunits. In addition to *moaD1*, *moaD2*, *moaE1*, *moaE2*, the *M. tuberculosis* genome also contains a *moaX* gene which encodes an MPT-synthase in which the MoaD and MoaE domains are located on a single polypeptide. In this study, we assessed the requirement for post-translational cleavage of MoaX for functionality of this novel, fused MPT synthase and attempted to establish a functional hierarchy for the various MPT-synthase encoding genes in *M. tuberculosis.*

**Results:**

Using a heterologous *Mycobacterium smegmatis* host and the activity of the MoCo-dependent nitrate reductase, we confirmed that *moaD2* and *moaE2* from *M. tuberculosis* together encode a functional MPT synthase. In contrast, *moaD1* displayed no functionality in this system, even in the presence of the MoeBR sulphurtransferase, which contains the rhodansese-like domain, predicted to activate MoaD subunits. We demonstrated that cleavage of MoaX into its constituent MoaD and MoaE subunits was required for MPT synthase activity and confirmed that cleavage occurs between the Gly82 and Ser83 residues in MoaX. Further analysis of the Gly81-Gly82 motif confirmed that both of these residues are necessary for catalysis and that the Gly81 was required for recognition/cleavage of MoaX by an as yet unidentified protease. In addition, the MoaE component of MoaX was able to function in conjunction with *M. smegmatis* MoaD2 suggesting that cleavage of MoaX renders functionally interchangeable subunits. Expression of MoaX in *E. coli* revealed that incorrect post-translational processing is responsible for the lack of activity of MoaX in this heterologous host.

**Conclusions:**

There is a degree of functional interchangeability between the MPT synthase subunits of *M. tuberculosis*. In the case of MoaX, post-translational cleavage at the Gly82 residue is required for function.

**Electronic supplementary material:**

The online version of this article (doi:10.1186/s12866-015-0355-2) contains supplementary material, which is available to authorized users.

## Background

*Mycobacterium tuberculosis* has an expanded genetic repertoire in several metabolic pathways, including those involved in energy metabolism, cell wall biosynthesis and DNA repair, a feature that is predicted to contribute to the enhanced metabolic responsiveness of this organism under stressful environmental conditions [1-4]. This capacity is illustrated by the first two steps of the pathway for the biosynthesis of molybdopterin cofactor (MoCo), which is synthesized through a multi-step pathway that requires the input of several proteins at each step [4-8]. Genes involved in MoCo biosynthesis and those encoding molybdoenzymes have been implicated in the pathogenesis of *M. tuberculosis* ([9] and reviewed in [4,10]). Molybdoenzymes facilitate redox reactions in carbon, nitrogen and sulphur metabolism in a diversity of eukaryotes and archea [8]. These findings, together with the proposed importance of this cofactor for pathogenesis, suggest that MoCo biosynthesis is a potentially interesting pathway for further understanding the lifestyle of the tubercle bacillus during infection.

**Figure 1:**
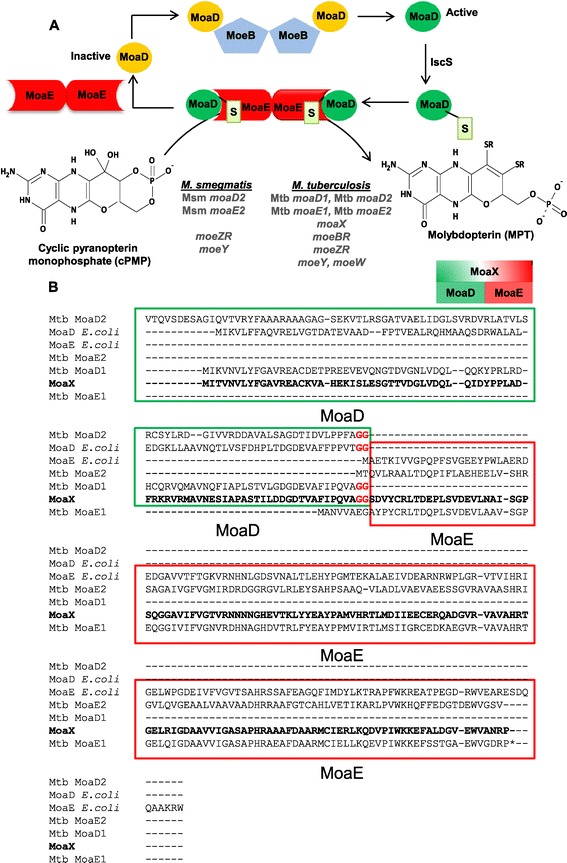
**Schematic representation of the proposed pathway for MPT formation from cPMP and sequence analysis of MoaX. (A)** MPT synthesis is catalyzed by the heterotetrameric MPT synthase, which is made up of two MoaD (Green-Active, Yellow-Inactive) and two MoaE (Red) subunits. S – Sulphur. The MPT synthase encoding genes in *M. smegmatis* and *M. tuberculosis* are shown to highlight the multiplicity of homologues in *M. tuberculosis*. Adapted from [14,17,22]. **(B)** Sequence alignment of *E. coli* MoaD (ECDH10B_0852) and MoaE (ECDH10B_0853), Mtb MoaD1 (Rv3112), Mtb MoaD2 (Rv0868c), Mtb MoaE1 (Rv3119), Mtb MoaE2 (Rv0866) and MoaX (Rv3323c) proteins. Conserved Gly residues are shown in red, these correspond to positions 81 and 82 in MoaX. The *M. tuberculosis* MoaX sequence is highlighted in bold; the MoaD component is shown in the green box and the MoaE component in the red box. The Alignment was generated using sequences obtained from Tuberculist (http://genolist.pasteur.fr/TubercuList/) and the Clustal Omega (http://www.ebi.ac.uk/Tools/msa/clustalo/) online alignment tool.

Molybdopterin (MPT) is formed in the second step of the pathway via a reaction which is catalyzed by the heterotetrameric MPT synthase, an enzyme comprising a pair of associated MoaD-MoaE heterodimers [6,8,11-13] (Figure 1A). The two sulphur molecules which are transferred to cyclic pyranopterin monophosphate (cPMP) are carried on the C-terminal glycine of MoaD as a thiocarboxylate. In order for the synthase to remain catalytically active, the sulfur on the C-terminus of MoaD must be regenerated in a series of steps (Figure 1A) [5,14]. MoeB is responsible for the ATP-dependent activation of the C-terminus of MoaD to form MoaD-adenylate [15], a reaction which requires the dissociation of MoaD from MoaE and formation of a MoeB-MoaD complex [16]. Studies in *Escherichia coli* have shown that the adenylated MoaD is then sulfurated by the cysteine desulphurase, IscS [17], an enzyme which acts as a sulfur donor in various reactions, including those required for the biosynthesis of biotin, thiamin and lipoic acid [18]. It was recently demonstrated that a rhodanese-like protein, YnjE, is required to direct IscS to MoCo biosynthesis in *E. coli* [19]. The formation of MPT proceeds via an intermediate carrying a single sulphur molecule and a terminal phosphate group, which remains tightly bound to the MoaE dimer. The conversion of the intermediate to MPT requires the dissociation of the uncharged MoaD and binding of a second thiocarboxylated MoaD [15].

In *M. tuberculosis*, the genes encoding MPT synthase subunits include *moaD1*, *moaD2*, *moaE1*, *moaE2* (referred to herein as Mtb *moaD1,* Mtb *moaD2,* Mtb *moaE1,* and Mtb *moaE2*) and *moaX*, where the latter encodes a fused molybdopterin synthase with MoaD- and MoaE-like domains located on a single polypeptide [4,20] (Figure 1B). In previous work, we demonstrated that Mtb *moaD1,* Mtb *moaD2* and *moaX* all contribute to molybdopterin biosynthesis in *M. tuberculosis* [21]. The presence of *moaX* in the genus *Mycobacterium* is restricted to those pathogenic mycobacteria that comprise the *M. tuberculosis* complex [21]. The demonstrated functionality of this atypical MPT-synthase-encoding gene suggests that it may serve a specific cellular function in pathogenesis [21]. However, the prevailing biochemical evidence pertaining to the catalytic mechanism of MPT synthase in *E. coli* [11,22] suggests that MoaX is likely to be non-functional as a single polypeptide for the following reasons: (I) In its active form, MoaD is thiocarboxylated at a C-terminal glycine (Gly) residue [5,22]. (II) During the formation of MPT, the sulfur atom on the C-terminus of MoaD is transferred to cPMP, bound in the substrate pocket of MoaE, which can accommodate both the C-terminus of MoaD as well as MPT or cPMP [22]; in this context, it is unlikely that the fused MoaX would accommodate this catalytic process. (III) MoaD, like ubiquitin, contains a C-terminal Gly-Gly motif which has been shown to be important for the function and stability of MPT synthase [23], and deletion, insertion or substitution mutants of these revealed that the terminal Gly residue (Gly81 in *E. coli* MoaD – corresponding to Gly82 in MoaX from *M. tuberculosis*, Figure 1B) and the penultimate Gly (Gly80 in *E. coli* MoaD – corresponding to Gly81 in MoaX from *M. tuberculosis*, Figure 1B) are essential for MPT synthase activity. (IV) Substitution of either Gly residue did not affect the ability to form a MoaD-MoaE heterodimer complex, although the G81A substitution in *E. coli* slowed complex formation by 60%, thus identifying this residue as essential for optimal MPT synthase function [23]. In MoaX, the corresponding Gly81-Gly82 residues are not located at the C-terminus but rather reside within the polypeptide chain (Figure 1B), thus suggesting that cleavage of MoaX into its MoaD and MoaE components is required in order to liberate the terminal glycine residues to mediate the above-mentioned functions.

*M. tuberculosis* contains two rhodanese-like proteins, MoeBR and MoeZR (previously annotated as MoeB2 and MoeB1, respectively), which are predicted to be involved in MoCo biosynthesis; both of these proteins are able to catalyze the sulphuration of MoaD1 and MoaD2 [24]. In prior work, we demonstrated that MoaD1 from *M. tuberculosis* was not functional in *M. smegmatis* [21], a finding which we ascribed to the lack of the corresponding rhodanese-like protein in *M. smegmatis*. In the present study, we investigate whether cleavage of MoaX is necessary for functionality of the resulting subunits. We also assess whether the presence of the rhodanese-like protein, MoeBR, is necessary for the function of *M. tuberculosis* MoaD homologues in *M. smegmatis*.

## Results

### Functional analysis of MPT synthase encoding genes from *M. tuberculosis*

**Figure 2:**
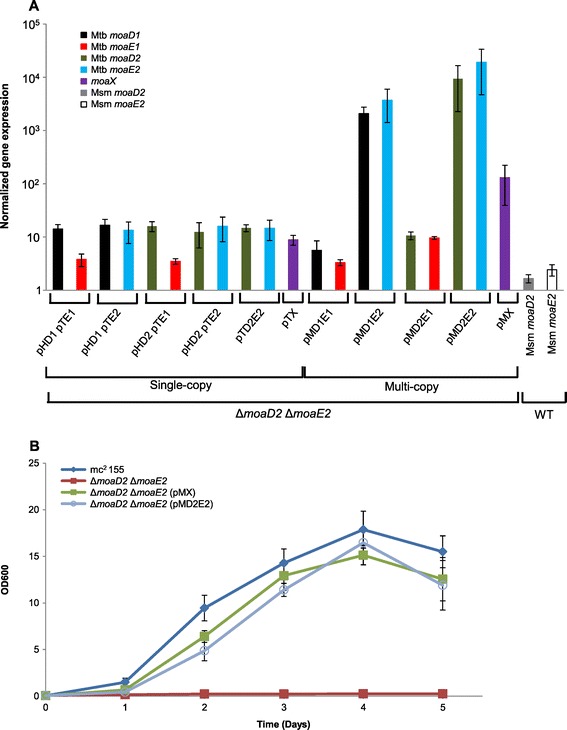
**Analysis of gene expression and MoCo biosynthesis in genetically complemented strains. (A)** Comparison of heterologous gene expression levels from integrating (single copy) versus episomal (multicopy) complementing vectors. The expression level of each gene was normalized against the expression of the *sigA* gene. In all cases, the data shown are representative of three independent biological replicates with standard errors of the mean. The expression of *moaD2* and *moaE2* in wild type (WT) *M. smegmatis* and the expression of *moaX*, when provided in single copy (pTX) or multicopy (pMX) was also assessed for comparison. **(B)** Nitrate assimilation in strains complemented with episomal vectors carrying different combinations of MPT-synthase-encoding genes. Strains were grown in MPLN medium supplemented with 10 mM Nitrate as the sole nitrate source.

The proteomes of *M. tuberculosis* and *M. smegmatis* are predicted to contain 7 and 20 molybdoenzymes respectively; included among these are respiratory and/or assimilatory nitrate reductase (NR) enzymes [4]. In *M. tuberculosis*, the membrane-bound NR, NarGHI, is the only NR present and it serves both respiratory and assimilatory functions [25]. In addition to NarGHI, *M. smegmatis* possesses an additional *narB*-encoded assimilatory NR, which is also predicted to be a molybdoenzyme [4]. In prior work, we developed an assay for MoCo availability in *M. smegmatis* which is based on measuring growth on nitrate as the sole nitrogen source via MoCo-dependent NR activity. We used this assay to confirm the essentiality of the sole MPT synthase subunit-encoding genes in this organism, designated herein as Msm *moaD2* and Msm *moaE2* [21] (Figure 1A), for MoCo biosynthesis. This growth-based assay thus provided a convenient means of evaluating the ability of various combinations of *M. tuberculosis moaD* and *moaE* genes to encode functional MPT synthase activity. We first tested if a double mutant of *M. smegmatis* lacking Msm *moaD2* and Msm *moaE2* (Δ*moaD2* Δ*moaE2*) carrying combinations of the Mtb *moaD1*, Mtb *moaD2*, Mtb *moaE1*, or Mtb *moaE2* genes, on vectors that integrated into the mycobacterial chromosome (Additional file 1: Table S1), displayed detectable NR activity. None of the combinations of *M. tuberculosis* genes tested was able to restore NR-dependent growth of the *M. smegmatis* Δ*moaD2* Δ*moaE2* mutant when provided in single copy via an integration vector (Table 1, Additional file 1: Figure S1). To test whether the lack of functional complementation was attributable to a low level of gene expression, we used qRT-PCR to assess gene expression levels in the various *M. smegmatis* recombinants. However, this analysis confirmed that in all cases, the *M. tuberculosis* genes were expressed at levels higher than the native Msm *moaD2* and Msm *moaE2* genes in wild type *M. smegmatis* (Figure 2A).

**Table 1:** **The ability of MPT-synthase encoding genes from** ***M. tuberculosis*** **to restore MoCo biosynthesis in the** ***M. smegmatis*** Δ***moaD2*** Δ***moaE2*** **mutant**

**MPT-synthase-encoding gene/s**	**Vector**	Functional complementation*
Mtb *moaD1* + Mtb *moaE1*	Integration	No
Mtb *moaD1* + Mtb *moaE2*	Integration	No
Mtb *moaD2* + Mtb *moaE1*	Integration	No
Mtb *moaD2* + Mtb *moaE2*	Integration	No
Mtb *moaD1*+ Mtb *moaE1*	Episomal	No
Mtb *moaD1*+ Mtb *moaE2*	Episomal	No
Mtb *moaD2*+ Mtb *moaE1*	Episomal	No
Mtb *moaD2* + Mtb *moaE2*	Episomal	Yes
*moaX*	Integration	Yes
*moaX*	Episomal	Yes
*moaXFL*	Episomal^§^	Yes
*moaXFL* ^*G81A*^	Episomal^§^	No
*moaXFL* ^*G82A*^	Episomal^§^	No

*As measured by the ability to restore growth of the *M. smegmatis* Δ*moaD2* Δ*moaE2* mutant on nitrate as sole nitrogen source. ^§^Carried on pFLAGEM, with a C-terminal 3 × FLAG-tag.

In prior work, we had demonstrated the functionality of Mtb *moaD2,* Mtb *moaE1* and Mtb *moaE2* through complementation of *M. smegmatis* mutants lacking either Msm *moaD2* or Msm *moaE2* by delivering the *M. tuberculosis* genes on an episomal plasmid [21], as opposed to an integrative vector. We therefore constructed synthetic operons carrying pairs of *M. tuberculosis moaD* and *moaE* homologs under the control of the mycobacterial *hsp60* promoter (Additional file 1: Table S2) and cloned these on an episomal plasmid. The resulting constructs were introduced into *M. smegmatis* Δ*moaD2* Δ*moaE2* and the resulting strains assessed for MoCo-dependent growth on nitrate as sole nitrogen source. As observed by Williams et al. [21]*,* episomally expressed *moaX* was able to restore MoCo biosynthesis in the Δ*moaD2* Δ*moaE2* mutant (Figure 2B). However, of the various combinations of *M. tuberculosis* genes tested, only the combination of Mtb *moaD2* and Mtb *moaE2* reconstituted MoCo biosynthesis, as evidenced by growth restoration of the double mutant (Figure 2B, Table 1). In contrast, none of the synthetic operon constructs carrying Mtb *moaD1* proved to be functional (Table 1) despite achieving gene expression levels comparable to, or higher than, the native Msm *moaD2* or Msm *moaE2* genes in wild type *M. smegmatis* (Figure 2A).

The lack of functionality of the *M. tuberculosis* Mtb *moaD2-*Mtb *moaE1* combination, when expressed episomally as a synthetic operon was surprising since these genes had been shown to individually complement single deletion mutants of *M. smegmatis* lacking either Msm *moaD2* or Msm *moaE2* [21]. We reasoned that this discrepancy might be due to differences in maturation of the MPT synthase or in sulphur metabolism between these organisms. Furthermore, the lack of functionality of Mtb *moaD1* in *M. smegmatis* was previously ascribed to the absence of *moeBR* in this organism which is proposed to preferentially interact with MoaD1 in *M. tuberculosis* since some mycobacterial strains have MoeZR and MoaD2 only, with no MoaD1 and MoeBR homologues [4,21,24]. In order to further assess the preferential requirement of MoeBR to activate Mtb MoaD1, *moeBR* from *M. tuberculosis* was expressed on an operon with Mtb *moaD1* and Mtb *moaE2* and delivered into the *M. smegmatis* Δ*moaD2* Δ*moaE2* mutant on an episomal vector. Expression analysis of the recombinant strain identified *moeBR* transcript at a level equivalent to that of Mtb *moaD1* (Additional file 1: Figure S2A). However, this strain was unable to grow on nitrate in minimal medium (Additional file 1: Figure S2B).

### MoaX is cleaved to yield two functional MPT synthase subunits

**Figure 3:**
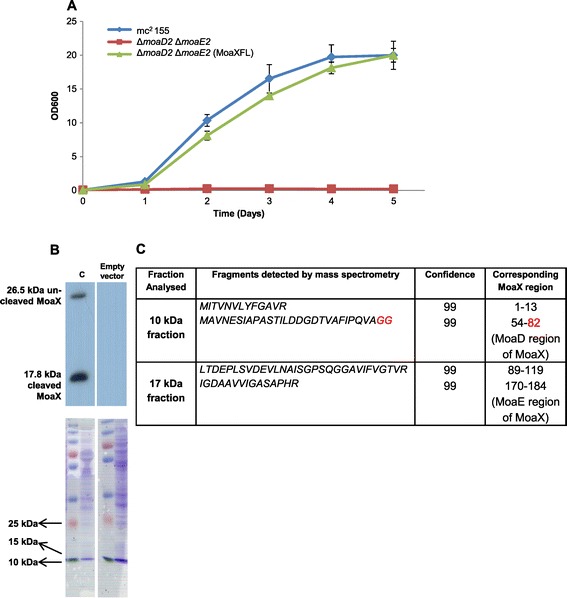
**FLAG-tagged MoaX is functional and cleaved in** ***M. smegmatis*** **.** **(A)** Nitrate assimilation in the Δ*moaD2* Δ*moaE2* mutant with a C-terminal FLAG-tagged derivative of *moaX*. In all cases, genes were expressed constitutively from the *tetO* operator. **(B)** Western blot analysis of protein extract from Δ*moaD2* Δ*moaE2* carrying C-terminally FLAG-tagged *moaX* showing cleavage of the fused MPT synthase at the Gly82 residue. Incorporation of the FLAG tag did not interrupt the function of MoaX, evidenced by the retained ability of the FLAG-tagged derivative to restore growth of Δ*moaD2* Δ*moaE2* in MPLN. **(C)** Peptide fragments of MoaX identified by mass spectrometry. Shown are two peptide fragments for each fraction. Red indicates the terminal Gly residues. Peptide reads all stopped at the Gly82 position, indicating cleavage at this point.

The active site of the MPT synthase is located within a pocket of MoaE and contains conserved C-terminal Gly residues of MoaD which are directly involved in enzyme activity [22]. We therefore hypothesized that MoaX might be post-translationally processed into MoaD and MoaE components in order to provide access to the residues Gly81 and Gly82 in the MoaD component of MoaX for further chemical modification. To test this, we first created a derivative of MoaX carrying a C-terminal FLAG-tag, termed MoaXFL. The MoaXFL derivative was fully proficient at restoring MoCo biosynthesis in the *M. smegmatis* Δ*moaD2* Δ*moaE2* mutant, confirming that the FLAG-tag did not disrupt MPT synthase activity (Figure 3A, Table 1). Western blot analysis of a cell extract from this strain, probing with an antibody directed to the FLAG-tag, revealed the presence of two major bands, which corresponded to the predicted sizes for full-length MoaXFL (26.5 kDa) and the FLAG-tagged MoaE domain of MoaX (17.8 kDa), respectively (Figure 3B). Peptide mass fingerprinting confirmed the presence of MoaE peptides in the 17.8 kDa band, with the terminal residue of one of the peptides corresponding to the Gly82 residue in MoaD (Figure 3C). No peptides that spanned the MoaD-MoaE junction were identified. These data confirmed cleavage at the Gly82 motif in MoaX to yield canonical MoaD and MoaE subunits. The presence of residual full length MoaX suggested that MoaX processing might be condition dependent. To further investigate this, we tested the extent of MoaX cleavage under different growth conditions, which included 7H9 broth or MPLN media. However, we found no difference in the extent of cleavage under these conditions (Additional file 1: Figure S3).

### The Gly81-Gly82 motif in MoaX is necessary for cleavage and function

**Figure 4:**
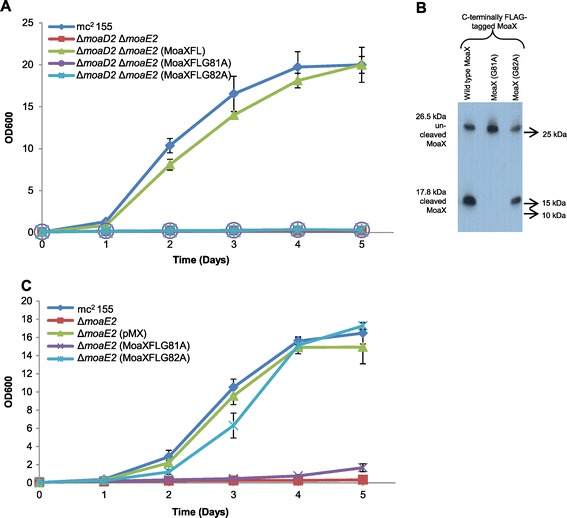
**Cleavage of MoaX is necessary for function. (A)** Nitrate assimilation in strains carrying FLAG-tagged mutated derivatives of MoaX. Shown are strains carrying either a G81A or G82A mutation. **(B)** Western blot analysis. An antibody directed at the FLAG-tag was used **(C)** Nitrate assimilation in the Δ*moaE2* single mutant of *M. smegmatis*. Growth curves were performed in MPLN medium with cell density measured over a period of 5 days. The G81A mutation abolished cleavage, whereas mutation G82A did not interfere with processing, but yielded a non-functional MoaD subunit. All data represent the result of three independent experiments.

As outlined above, the two terminal Gly residues in MoaD have been identified as important for MPT synthase activity in *E. coli* [23]. Sequence alignment of MoaX with Mtb MoaD1 and Mtb MoaD2 as well as with *E. coli* MoaD reveals that these Gly residues, Gly81 and Gly82 (positions in the *M. tuberculosis* enzymes), are conserved in MoaX (Figure 1B). In order to assess whether these residues are important for MoaX function and/or cleavage, site-directed mutagenesis of the *moaXFL* gene was carried out to individually replace each Gly with an Ala. MoCo biosynthesis was then assessed in strains carrying the corresponding mutant *moaXFL*^*G81A*^ or *moaXFL*^*G82A*^ alleles. Unlike wild type MoaXFL, the mutant derivatives, MoaXFL^G81A^ and MoaXFL^G82A^, were unable to restore MoCo biosynthesis in the MPT-synthase deficient mutant of *M. smegmatis* (Figure 4A, Table 1). In addition to being essential for functionality, Western blot analysis revealed that Gly81 is also required for cleavage of MoaX since no discernible cleavage was detected for the MoaXFL^G81A^ mutant protein (Figure 4B). In contrast, while essential for function, Gly82 did not appear to be required for MoaX processing as evidenced by the presence of a 17.8 kDa MoaE band in the Western blot analysis of the strain expressing MoaXFL^G82A^ (Figure 4B).

The mutated Gly81/Gly82 residues form part of the MoaD component of MoaX, suggesting that once cleaved, the MoaE component of the enzyme would remain active. In order to test this, the vectors expressing MoaXFL^G81A^ or MoaXFL^G82A^ were introduced into the *M. smegmatis* Δ*moaE2* single mutant and the recombinant strains assessed for growth on nitrate as sole nitrogen source. MoCo biosynthesis was restored in the strain expressing MoaX^G82A^ (Figure 4C), which confirmed that the Mtb MoaE subunit released through cleavage of MoaX can associate with Msm MoaD2 to reconstitute a functional, chimeric MPT synthase. In contrast, the Δ*moaE2* strain expressing the non-functional, un-cleavable MoaXFL^G81A^ was severely attenuated for growth on nitrate as sole nitrogen source (Figure 4C), providing further evidence that MoaX cleavage is indeed required for the function of the resulting MoaE subunit.

**Figure 5:**
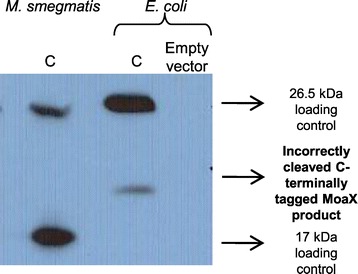
**Western blot analysis of MoaXFL in** ***E. coli*** **.** Included as a positive control is protein extract from *M. smegmatis* expressing *moaX* from the same system. The correct 26.5 kDa band corresponding to un-cleaved MoaX is observed in both organisms. However, the 17.8 kDa cleavage product corresponding to the MoaE component of MoaX is not observed in *E. coli,* which displays a larger (~20 kDa) band.

Finally, a recent study revealed that *M. tuberculosis moaX* was unable to complement *E. coli moaD* or *moaE* single mutants [24]. The authors of this study hypothesized that this was due to the lack of MoaX cleavage machinery in *E. coli*. We addressed this question by Western blot analysis of protein extracts from *E. coli* cells expressing MoaXFL. Full-length MoaXFL as well as a minor band of higher molecular weight than expected for the MoaE subunit were observed in the Western blot. However, in contrast to the *M. smegmatis* control, a band corresponding to the FLAG-tagged MoaE component of MoaX was not observed in the *E. coli* extract (Figure 5).

### Discussion

*M. tuberculosis* has a multiplicity of MPT-synthase-encoding genes which all contribute to MoCo biosynthesis [4]. The observation that chimeras of MPT synthase from different combinations of human and *E. coli* subunits display varying catalytic efficiencies [26], together with the demonstrated functionality of the Mtb *moaD1*, Mtb *moaD2*, Mtb *moaE1*, and Mtb *moaE2* genes [21], raised the possibility that different isoforms of MPT synthase might occur in *M. tuberculosis*. However, only a combination of Mtb *moaD2* and Mtb *moaE2*, when expressed at high levels from an episomal plasmid in *M. smegmatis*, was able to complement the conditional growth phenotype of the Δ*moaD2* Δ*moaE2* mutant; none of the other combinations of Mtb *moaD* and *moaE* homologues that were tested formed a functional MPT synthase. The expression levels of complementing genes in strains carrying Mtb *moaE1*, either on integrative or episomal vectors, were all relatively low when compared to those carrying Mtb *moaE2*, suggesting that an expression threshold may be required in order to achieve functional complementation in the heterologous host. Importantly, however, *moaX* complemented the growth phenotype of the double mutant when expressed from an integrative vector at a comparably low level, suggesting that a factor/s other than expression level was responsible for the differential complementing abilities of the Mtb *moaD*/*moaE* combinations.

In *E. coli*, MoeB adenylates MoaD which is subsequently sulfurated by IscS and a rhodanese-like protein, YnjE, to generate the thiocarboxylated form of MoaD required for MPT formation [17,19]. In *M. tuberculosis*, both MoeBR and MoeZR contain a rhodanese-like domain and are capable of sulfur transfer in vitro to Mtb MoaD1 and Mtb MoaD2 [24]. However, the amino acid sequence of Mtb MoaD2 is more closely related to the sulphur carrier protein, CysO, than to Mtb MoaD1. This observation, together with the fact that CysO is a preferential substrate for MoeZR, has led to the hypothesis that Mtb MoaD1 is preferentially activated by MoeBR, while Mtb MoaD2 is activated by MoeZR [24]. This is supported by the fact that other mycobacteria, including *Mycobacterium avium* 104, *Mycobacterium marinum* and *Mycobacterium ulcerans*, contain MoaD2 and MoeZR, but not MoaD1 and MoeBR homologues [9,21]. Importantly, both Mtb MoaD1 and MoeBR are located on a mobile genetic cluster that was acquired by horizontal gene transfer [27] thus further supporting the notion that MoeBR preferentially activates Mtb MoaD1. *M. smegmatis* possesses a single *moaD* gene (*moaD2*) and *moeZR* suggesting that the lack of functionality of Mtb MoaD1 in this organism could be attributable to differences in the complement of enzymes with a rhodanese-like domain. However, inclusion of *moeBR* in an expression cassette carrying Mtb *moaD1* and Mtb *moaE2* did not result in functional complementation of the MoCo biosynthesis defect of the Δ*moaD2* Δ*moaE2* mutant. This suggests that the lack of functionality for Mtb *moaD1* may be due to other reasons such as differences in the complement sulphur carrier proteins, and/or in cysteine biosynthesis.

In *E. coli*, CysO is involved in cysteine biosynthesis [24], which highlights a role for MoeZR in both amino acid and MoCo biosynthesis, thus linking these metabolic pathways. Cysteine has been implicated in *M. tuberculosis* pathogenesis by providing protection against reactive oxygen/nitrogen intermediates [28]; this also suggests a role for MoeZR under these conditions, which is supported by the up-regulation of *cysM, cysO* and *moeZR* in *M. tuberculosis* under oxidative stress [29]. The mycobacterial sulfur source for MoCo biosynthesis remains unknown but is most likely L-cysteine [24,30], in which case, an L-cysteine desulphurase such as IscS would transfer sulfur to MoeZR. In *E. coli,* IscS is implicated in iron-sulfur cluster homeostasis [31] and may have a similar role in mycobacteria which, through an interaction with MoeZR, would link iron-sulfur cluster homeostasis with the second step of MoCo biosynthesis. It is therefore reasonable to conclude that disruptions in the MPT synthase step of the pathway would not only affect MoCo biosynthesis but might also disturb cysteine biosynthesis and sulfur homeostasis. Consistent with this is the large number of transposon mutants of *M. tuberculosis* which map to the first two steps of the MoCo biosynthesis pathway and are defective for intracellular growth or survival in animals [32-37].

**Figure 6:**
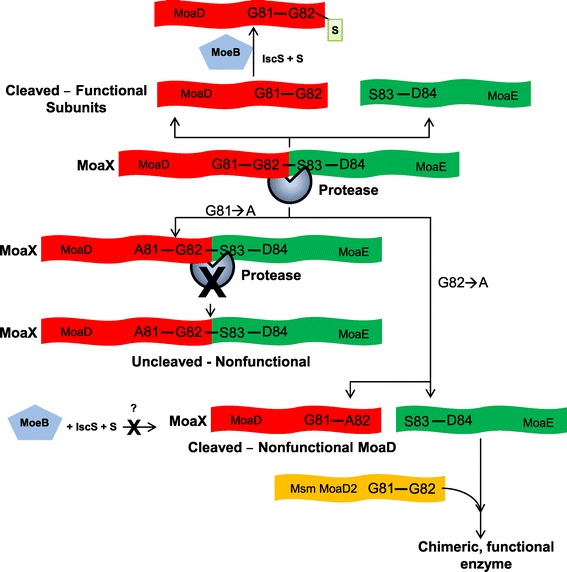
**The role of the Gly81-Gly82 residues in MoaX.** The Gly81 and Gly82 residues in MoaX are required for functionality. Mass spectrometry analysis of MoaX cleavage products confirmed that proteolytic processing occurs between the Gly82-Ser83 motifs to yield two functional MPT-synthase subunits. Mutation of Gly81 abrogates processing, yielding a non-functional, uncleaved derivative of MoaX suggesting that this residue is important for recognition/binding of the protease. In contrast, the G82→A mutation does not affect cleavage but the resulting MoaD-like subunit is not functional due to the absence of the terminal glycine, which is required for activation of MoaD by MoeB. However, the MoaE subunit that results from cleavage of this mutant protein can combine with the Msm MoaD2 to reconstitute a functional enzyme.

Structural analysis of the *E. coli* MPT synthase revealed that the essential terminal Gly residue of MoaD is embedded in a pocket of MoaE where the sulphur transfer reaction occurs [11]. Considering this domain organization and catalysis sequence, it was unclear how the single polypeptide, encoded by *moaX* would be able to function. In this study, we demonstrated cleavage of MoaX and further studied the role of the Gly81-Gly82 residues; a model that describes our findings is shown in Figure 6. The Gly81 residue is required for proteolytic processing and the inability of the MoaXFL^G81A^ mutant protein to restore MoCo biosynthesis confirmed that cleavage of MoaX is necessary for catalysis. This is corroborated by the ability of the non-functional but cleaved MoaXFL^G82A^ mutant protein to restore MPT synthase activity in the Δ*moaE2* mutant, which retains a functional Msm MoaD2. In this case, MoCo biosynthesis is restored through the activity of a chimeric Msm MoaD2- Mtb MoaE enzyme, confirming that cleavage of MoaX releases functionally interchangeable subunits. However, we cannot rule out the possibility that mutation of the Gly81 residue in the un-cleaved protein may also contribute to lack of function. The identification of residual full-length MoaX for all cases in which cleavage was observed suggests that in *M. smegmatis*, the cleavage process is either slow or subject to regulation.

Finally, a recent study by Voss et al. [24], established that MoaX was not functional in *E. coli* and was unable to interact with either MoeBR or MoeZR from *M. tuberculosis*. The authors speculated that this was due to the lack of the MoaX cleavage machinery in *E. coli* as opposed to an inherent inability of these proteins to interact. In this study, we demonstrated that some cleavage of MoaX does occur when constitutively expressed in *E. coli* DH5α*;* however, the cleavage product was larger than expected, which might explain the inability of MoaX to function in *E. coli*.

## Conclusions

In conclusion, we have demonstrated that there is a degree of functional interchangeability between the MPT synthase subunits of *M. tuberculosis*. In the case of MoaX, post-translational cleavage at the Gly82 residue is required for functionality of this novel MPT synthase.

## Methods

### Bacterial strains and culture conditions

The bacterial strains used and generated during this study are listed in Additional file 1: Table S1. *E. coli* strains were grown in Luria Bertani liquid medium (LB) or solid medium (LA) supplemented with the appropriate antibiotics at concentrations of 200 μg/ml Hygromycin (Hyg) and 50 μg/ml Kanamycin (Kan). *M. smegmatis* strains were grown in Middlebrook 7H9 liquid medium (Difco) supplemented with Middlebrook oleic acid-albumin-dextrose-catalase (OADC) enrichment (Difco), 0.2% glycerol and 0.05% Tween80, with shaking, or on Middlebrook 7H10 solid medium (Difco) supplemented with 0.085% NaCl, 0.2% glucose and 0.5% glycerol. Media for *M. smegmatis* growth was supplemented with antibiotics at concentrations of 50 μg/ml Hyg and/or 25 μg/ml Kan where appropriate. Nitrate assimilation to measure MoCo biosynthesis was carried out as previously described in [21]. Briefly, pre-cultures were washed and inoculated into modified *Mycobacterium phlei* minimal medium (MPLN), which was modified by excluding asparagine and substituting with 10 mM sodium nitrate, in a final volume of 10 ml to an optical density at 600 nm (OD_600_) of 0.05. Cultures were monitored for 5 days with OD_600_ readings recorded daily. All cultures were incubated at 37°C, with shaking at 115 rpm.

### Complementation vector constructions

The multi-copy episomal vectors (pTBD1, pTBD2, pTBE1 and pTBE2), carrying each of the *M. tuberculosis moaD* and *moaE* homologues, provided by Dr. M. Williams [21], were used for the construction of vectors in the present study. Using the restriction enzymes *Bgl*II and *Pvu*I, the genes, together with their *hsp60* promoters were excised from these vectors. The integrating vectors, pTT1B and pHINT, were linearized with *Sca*I. Mtb *moaE1* and Mtb *moaE2* fragments were ligated to linearized pTT1B to generate the integrating vectors, pTE1 and pTE2, respectively. The Mtb *moaD1* and Mtb *moaD2* genes were similarly incorporated into pHINT, forming the vectors pHD1 and pHD2, respectively. An integrating vector carrying *moaX* was similarly constructed by excising the gene together with its *hsp60* promoter from pMoaX [21] with *Sac*II and ligating it to the *Sca*I-linearized pTT1B vector, to generate the integrating vector, pTX. Details of the vectors are provided in Additional file 1: Table S2.

Episomal vectors carrying different combinations of the *M. tuberculosis moaD* and *moaE* homologues were constructed using a PCR cloning strategy. This strategy allowed for the introduction of Mtb *moaD1* and Mtb *moaD2* upstream of both Mtb *moaE1* and Mtb *moaE2* carried on episomal vectors, and facilitated the operonic expression of two genes driven off a single *hsp60* promoter. The primers, mD1F and mD1R, were used to amplify Mtb *moaD1*, while mD2F and mD2R were used to amplify Mtb *moaD2*. The purified PCR products as well as the vectors pTBE1 and pTBE2 were digested with *Pst*I and *Hind*III to allow for directional cloning. The digested fragments were ligated together in different combinations to generate the episomal vectors pMD1E1, pMD1E2, pMD2E1 and pMD2E2.

The primers moeBRF and moeBRR were used to amplify *M. tuberculosis moeBR* from genomic DNA. The 1178 bp PCR product was digested with *Pac*I while the vector pMD1E2 was digested with *Pst*I. Both fragments were blunted and ligated to generate the plasmid BRD1E2. Details of primers are provided in Additional file 1: Table S3.

### FLAG-tagging of proteins

The FLAG-tag vector pFLAGEM, carrying a 3 × FLAG-tag [38], was generated by ligating a double stranded linker with overhangs compatible to the *Sph*I and *Hind*III sites, to replace the *Sph*I to *Hind*III fragment of pSE100. The linker was designed with a nucleotide bias towards increased G + C content, while retaining the same peptide sequence. Placement of *Bsr*GI and *Acc*65I sites allows for cloning the same PCR fragment to create either N- or C-terminally FLAG-tagged proteins. The primers moaXF and moaXR (Additional file 1: Table S3) were used to amplify *moaX*. The 679 bp PCR product was digested with *Bsr*GI and *Bsi*WI prior to ligation. The digested PCR fragment was ligated with *Bsr*GI digested pFLAGEM for incorporation of the tag at the C-terminus of MoaX to generate pMoaXFL (Additional file 1: Table S2).

### Mutagenesis vector constructions

The Megaprimer method [39] was used to introduce two independent point mutations into *moaX*. The primers used are listed in Additional file 1: Table S3. The mutated reverse primer moaXga1R was used to incorporate the 242G→C point mutation into *moaX* which resulted in a Gly→Ala change at position 81 of MoaX. A second mutated reverse primer moaXga2R was used to incorporate the 245G → C point mutation into *moaX* which resulted in a Gly → Ala change at position 82 of MoaX. Full-length mutated *moaX* genes were cloned into pFLAGEM in the same manner as the wild type gene. All of the vectors generated in this study were confirmed by restriction analysis and sequencing.

### Protein extractions

Cultures were grown in 50 or 100 ml of 7H9 medium to an OD_600_ of 1–1.4 and harvested by centrifugation at 2 360 × *g* for 10 min. Pellets were then re-suspended in B-PER (Fischer Scientific) cocktail solution (250 μl/50 ml culture) and either stored at - 80°C or lysed immediately. For lysis, cells were transferred to Lysing Matrix B (IEPSA) tubes which contain 0.1 μm silica beads for the mechanical shearing of cells. Cells were lysed by ribolysing the tubes in the FastPrep Savant FP-120 Ribolyser for 20 sec at speed 6 with three repeats and 5 min incubations on ice between each run. The tubes were spun down at 12 470 × *g* for 10 sec to pellet the cell debris and silica beads. The supernatant was then transferred to clean tubes and centrifuged at 12 470 × *g* for 5 min to separate the soluble and insoluble protein fractions. MoaX was predominantly observed in the soluble fraction. For the extraction of protein from *E. coli* cells, 10-20-ml cultures were grown in LB with the appropriate antibiotics and harvested by centrifugation at 2 360 × *g* for 10 min. Cell pellets were then re-suspended in 250–500 μl of B-PER cocktail and incubated at room temperature for 10 min. Cell debris was collected by centrifugation at 12 470 × *g* for 5 min and the supernatant was used for downstream processes.

### Western blot analysis

The primary antibody used for all Western blots in this study was ANTI-FLAG M2® Monoclonal Antibody, mouse-purified IgG (Sigma) at a final concentration of 10 μg/ ml and the secondary antibody used was Rabbit Anti-Mouse IgG, Peroxidase Conjugate (Sigma) at a dilution of 1:25 000–40 000. Membranes were incubated with the primary antibody for 1 hr at room temperature or at 4°C overnight with gentle agitation. This was followed by three washes in TBST (Tris-Buffered Saline and Tween 20) for 5 min each. Incubations with the secondary antibody were carried out for 1 hr at room temperature with gentle shaking. This was followed by five wash steps of 5 min each with TBST at room temperature. Chemiluminescent Peroxidase Substrate (CPS) Reagent (Sigma) was then added and the membrane was exposed to X-ray film.

### Protein identification

The nanoLC-MSMS based peptide sequencing method was used to identify protein fragments excised from Coomasie blue-stained gels. This analysis was performed at the Council for Scientific and Industrial Research (CSIR) in Pretoria, South Africa, Dr Stoyan Stoychev. Mass spectrometry data was analyzed using ProteinPilot™ Software 3.0.

### Quantitative Real-Time PCR

Cultures for RNA extraction were grown in 7H9 medium to an OD_600_ of 0.5 at which point cells were harvested by centrifugation at 2 360 × *g* for 10 min. RNA was extracted using the NucleoSpin RNA II Kit (Support Protocol 5.3) as per manufacturer’s instructions. For complete disruption of the mycobacterial cell wall, three rounds of ribolyzing were performed at 4.5 for 45 s, with cooling on ice for 2 mins between each round. RNA conversion to cDNA was performed using 1 μg RNA and SuperScript III (Life Technologies) as per the manufacturer’s instructions. RT PCR was performed using the synthesized cDNA and Evagreen Supermix (Biorad) as per the manufacturers’ instructions. The primers used for qRT PCR are listed in Additional file 1: Table S4. Expression of each gene was normalized against the *sigA* expression level.

## Additional file

Additional file 1: Figure S1. Growth curve of *M. smegmatis* Δ*moaD2* Δ*moaE2* complemented with different combinations of Mtb *moaD1,* Mtb *moaD2,* Mtb *moaE1* and Mtb *moaE2* carried on integrating vectors. Growth curves were carried out in MPLN medium, supplemented with 10 mM sodium nitrate with optical density readings taken daily for 5 days. The data are an average of at least three independent experiments. **Figure S2.** MoeBR does not confer functionality with MoaD1 homologues. (A) Gene expression analysis of *M. smegmatis* double mutant strain complemented with Mtb *moaD1,* Mtb *moaE2* and *moeBR*. The expression of each gene was normalized against *sigA* expression. Data were generated from three independent experiments. (B) Nitrate assimilation in *M. smegmatis* strains. Growth curves were carried out in MPLN, supplemented with 10 mM, sodium nitrate. The growth curve is an average of four independent experiments with standard errors. **Figure S3.** Cleavage of MoaX under different growth conditions. Strains carrying pMoaXFL were transformed with pMC1s, which carries the TetR repressor. In this case, the addition of anhydrotetracycline induces expression of *moaXFL*. Cleavage was tested in standard 7H9 broth and in MPLN media (A) MoaX cleavage in mc^2^155 (B) MoaX cleavage in Δ*moaD2* Δ*moaE2*:: pMC1s (pMoaXFL). **Table S1.** *M. smegmatis* strains used and generated during this study. **Table S2.** Plasmids used and generated during this study. **Table S3.** Primers used for construction of plasmids **Table S4.** Primers used for q(RT)PCR analysis.

## Abbreviations

Ala: Alanine
cPMP: Cyclic pyranopterin monophosphate
Gly: Glycine
MPLN: Modified *M. phlei*media
Mo: Molybdenum
MPT: Molybdopterin
MoCo: Molybdenum cofactor
NR: Nitrate reductase
Ser: Serine
S: Sulphur
