# Supplementary material for: Cleavage of the moaX-encoded fused molybdopterin synthase from Mycobacterium tuberculosis is necessary for activity
Source: BMC Microbiol. 2015 Feb 6;15(1):22. doi: 10.1186/s12866-015-0355-2 (PMC4326299; doi:10.1186/s12866-015-0355-2)
Supplement: Additional file 1: Figure S1. — Growth curve of M. smegmatis ΔmoaD2 ΔmoaE2 complemented with different combinations of Mtb moaD1, Mtb moaD2, Mtb moaE1 and Mtb moaE2 carried on integrating vectors. Growth curves were carried out in MPLN medium, supplemented with 10 mM sodium nitrate with optical density readings taken daily for 5 days. The data are an average of at least three independent experiments. Figure S2. MoeBR does not confer functionality with MoaD1 homologues. (A) Gene expression analysis of M. smegmatis double mutant strain complemented with Mtb moaD1, Mtb moaE2 and moeBR. The expression of each gene was normalized against sigA expression. Data were generated from three independent experiments. (B) Nitrate assimilation in M. smegmatis strains. Growth curves were carried out in MPLN, supplemented with 10 mM, sodium nitrate. The growth curve is an average of four independent experiments with standard errors. Figure S3. Cleavage of MoaX under different growth conditions. Strains carrying pMoaXFL were transformed with pMC1s, which carries the TetR repressor. In this case, the addition of anhydrotetracycline induces expression of moaXFL. Cleavage was tested in standard 7H9 broth and in MPLN media (A) MoaX cleavage in mc2155 (B) MoaX cleavage in ΔmoaD2 ΔmoaE2:: pMC1s (pMoaXFL). Table S1. M. smegmatis strains used and generated during this study. Table S2. Plasmids used and generated during this study. Table S3. Primers used for construction of plasmids Table S4. Primers used for q(RT)PCR analysis. [file 12866_2015_355_MOESM1_ESM.pdf]

Cleavage of the *moaX*-encoded fused molybdopterin synthase from *Mycobacterium tuberculosis*is necessary for activity

Nicole C. Narrandes[1], Edith Erika Machowski,[1] Valerie Mizrahi[2] and Bavesh D. Kana[1*]

Supplementary Information

[1]DST/NRF Centre of Excellence for Biomedical TB Research, Faculty of Health Sciences, University of the Witwatersrand, National Health Laboratory Service, Johannesburg, South Africa

[2] MRC/NHLS/UCT Molecular Mycobacteriology Research Unit and DST/NRF Centre of Excellence for Biomedical TB Research, Institute of Infectious Disease & Molecular Medicine and Division of Medical Microbiology, University of Cape Town, South Africa

Nicole.Narrandes@nhls.ac.za; Edith.Machowski@nhls.ac.za; Valerie.Mizrahi@uct.ac.za

*For correspondence: Bavesh Davandra Kana, DST/NRF Centre of Excellence for Biomedical TB Research, School of Pathology, Faculty of Health Sciences, University of the Witwatersrand and the National Health Laboratory Service, P.O. Box 1038, Johannesburg, 2000, South Africa. Email: bavesh.kana@nhls.ac.za. Telephone: +27 11 489 9030, Fax: +27 11 489 9397

Supplementary Tables

Table S1: List of *Mycobacterium smegmatis* strains used and generated during this study.

**Name**	**Description**	**Source/ reference**
<u>*M. smegmatis*</u>		
mc^{2}155	*ept-1* (efficient plasmid transformation) mutant of mc^{2}6	[1]
Δ*moaE2*	Derivative of mc^{2}155 carrying an unmarked deletion in the Msm *moaE2* gene	[2]
Δ*moaE2* (pMX)	Derivative of *M. smegmatis* Δ*moaE2* carrying an episomal plasmid expressing *moaX* from the *hsp60* promoter; Hyg^r	[2]
Δ*moaD2* Δ*moaE2*	Derivative of *M. smegmatis* Δ*moaE2* carrying an unmarked deletion in the Msm *moaD2* gene	[2]
Δ*moaD2* Δ*moaE2* (pMX)	Derivative of *M. smegmatis* Δ*moaD2* Δ*moaE2* carrying an episomal plasmid expressing *moaX* from the *hsp60* promoter; Hyg^r	[2]
Δ*moaD2* Δ*moaE2*:: pTX	Derivative of *M. smegmatis* Δ*moaD2* Δ*moaE2* carrying an integrating plasmid expressing *moaX* from the *hsp60* promoter; Kan^r	This work
Δ*moaD2* Δ*moaE2*::pHD1 pTE1	Derivative of *M. smegmatis* Δ*moaD2* Δ*moaE2* with integrating plasmids pHD1* and pTE1* expressing Mtb *moaD1* and Mtb *moaE1* respectively from the *hsp60* promoter; Hyg^r, Kan^r	This work
Δ*moaD2* Δ*moaE2*::pHD1 pTE2	Derivative of *M. smegmatis* Δ*moaD2* Δ*moaE2* with integrating plasmids pHD1 and pTE2 expressing Mtb *moaD1* and Mtb *moaE2* respectively from the *hsp60* promoter; Hyg^r, Kan^r	This work
Δ*moaD2* Δ*moaE2*::pHD2 pTE1	Derivative of *M. smegmatis* Δ*moaD2* Δ*moaE2* with integrating plasmids pHD2 and pTE1 expressing Mtb *moaD2* and Mtb *moaE1* respectively from the *hsp60* promoter; Hyg^r, Kan^r	This work

Δ*moaD2* Δ*moaE2*::pHD2 pTE2	Derivative of *M. smegmatis* Δ*moaD2* Δ*moaE2* with integrating plasmids pHD2 and pTE2 expressing Mtb *moaD2* and Mtb *moaE2* respectively from the *hsp60* promoter; Hyg^r, Kan^r	This work
Δ*moaD2* Δ*moaE2*::pTD2E2	Derivative of *M. smegmatis* Δ*moaD2* Δ*moaE2* with integrating plasmid pTD2E2 expressing Mtb *moaD2* and Mtb *moaE2* from a single upstream *hsp60* promoter; Kan^r	This work
ΔmoaD2 ΔmoaE2 (pMD1E1)	Derivative of *M. smegmatis* Δ*moaD2* Δ*moaE2* with episomal plasmid pMD1E1 expressing Mtb *moaD1* and Mtb *moaE1* from a single upstream *hsp60* promoter; Hyg^r	This work
Δ*moaD2* Δ*moaE2* (pMD1E2)	Derivative of *M. smegmatis* Δ*moaD2* Δ*moaE2* with episomal plasmid pMD1E2 expressing Mtb *moaD1* and Mtb *moaE2* from a single upstream *hsp60* promoter; Hyg^r	This work
Δ*moaD2* Δ*moaE2* (pMD2E1)	Derivative of *M. smegmatis* Δ*moaD2* Δ*moaE2* with episomal plasmid pMD2E1 expressing Mtb *moaD2* and Mtb *moaE1* from a single upstream *hsp60* promoter; Hyg^r	This work
Δ*moaD2* Δ*moaE2* (pMD2E2)	Derivative of *M. smegmatis* Δ*moaD2* Δ*moaE2* with episomal plasmid pMD2E2 expressing Mtb *moaD2* and Mtb *moaE2* from a single upstream *hsp60* promoter; Hyg^r	This work
Δ*moaD2* Δ*moaE2* (pMBRD1E2)	Derivative of *M. smegmatis* Δ*moaD2* Δ*moaE2* with episomal plasmid pMBRD1E2 expressing *M. tuberculosis moeBR*, Mtb *moaD1* and Mtb *moaE2* from a single upstream *hsp60* promoter; Hyg^r	This work
Δ*moaD2*Δ*moaE2* (pMoaXFL)	Derivative of Δ*moaD2* Δ*moaE2* with episomal plasmid MoaXFL expressing C-terminally 3×FLAG-tagged *moaX* under the control of the *tetO*; Hyg^r	This work
Δ*moaD2*Δ*moaE2* (pMoaXFLG81A)	Derivative of Δ*moaD2* Δ*moaE2* with episomal plasmid MoaXFLG81A expressing mutated C-terminally 3×FLAG-tagged *moaX* under the control of the *tetO*; Hyg^r	This work
Δ*moaD2*Δ*moaE2* (pMoaXFLG82A)	Derivative of Δ*moaD2* Δ*moaE2* with episomal plasmid MoaXFLG82A expressing mutated C-terminally 3×FLAG-tagged *moaX* under the control of the *tetO*; Hyg^r	This work
Δ*moaE2*	Derivative of Δ*moaE2* with episomal plasmid MoaXFLG81A expressing mutated C-terminally 3×FLAG-tagged *moaX*	This work

(pMoaXFLG81A)	under the control of the *tetO*; Hyg^r	
Δ*moaE2* (MoaXFLG82A)	Derivative of Δ*moaE2* with episomal plasmid MoaXFLG82A expressing mutated C-terminally 3×FLAG-tagged *moaX* under the control of the *tetO*; Hyg^r	This work
Δ*moaD2* Δ*moaE2::*pMC1s (pMoaXFL)	Derivative of Δ*moaD2* Δ*moaE2* with integrating vector pMC1s expressing the *tet* repressor, TetR and carrying the episomal plasmid pMoaXFL expressing C-terminally 3×FLAG-tagged *moaX* under the control of the *tetO*; Kan^r, Hyg^r	This work
mc^2155::pMC1s (pMoaXFL)	Derivative of $mc^2155$with integrating vector pMC1s expressing the *tet* repressor, TetR and carrying the episomal plasmid pMoaXFL expressing C-terminally 3×FLAG-tagged *moaX* under the control of the *tetO*; Kan^r, Hyg^r	This work

* pH- and pT- plasmids integrate at the *attB* (tRNA for glycine) and pTweety (tRNA for lysine) sites respectively

Table S2: List of plasmids used and generated during this study.

**Name**	**Description**	**Source/ Reference**
Plasmids		
pTTP1B	*E. coli*-*Mycobacterium* integrating shuttle vector Kan^r – integrates at the tRNA for lysine	[3]
pHINT	*E. coli*-*Mycobacterium* integrating shuttle vector; Amp^r, Hyg^r	[4]
pSE100	*E. coli*-*Mycobacterium* shuttle vector carrying the tetracycline operator (*tetO*)	[5]
pMC1s	L5-based integration vector carrying P_{smyc}-*tetR*; Kan^r	[5]

pMhsp60	Mycobacterial expression vector constructed by deleting the region between the *Nco*I and *Pvu*II sites in pUAB300; Hyg^r	[2]
pTBD1	Derivative of pMhsp60 carrying Mtb *moaD1* expressed under control ofthe *hsp60* promoter; Hyg^r	[2]
pTBD2	Derivative of pMhsp60 carrying Mtb *moaD2* expressed under control of the*hsp60* promoter; Hyg^r	[2]
pTBE1	Derivative of pMhsp60 carrying Mtb *moaE1* expressed under control of the*hsp60* promoter; Hyg^r	[2]
pTBE2	Derivative of pMhsp60 carrying Mtb *moaE2* expressed under control of the *hsp60* promoter; Hyg^r	[2]
pHD1	Derivative of pHINT carrying Mtb *moaD1* expressed under control of the *hsp60* promoter; Hyg^r	This work
pHD2	Derivative of pHINT carrying Mtb *moaD2* expressed under control of the *hsp60* promoter; Hyg^r	This work
pTE1	Derivative of pTT1B carrying Mtb *moaE1* expressed under control of the *hsp60* promoter; Kan^r	This work
pTE2	Derivative of pTT1B carrying Mtb *moaE2* expressed under control of the *hsp60* promoter; Kan^r	This work
pTX	Derivative of pTT1B carrying Mtb *moaX* expressed under control of the *hsp60* promoter; Kan^r	This work
pMD1E1	Derivative of pMhsp60 carrying Mtb *moaD1* and Mtb *moaE1*expressed as an operon under control of the *hsp60* promoter; Hyg^r	This work
pMD1E2	Derivative of pMhsp60 carrying Mtb *moaD1* and Mtb *moaE2* expressed as an operon under control of the *hsp60* promoter; Hyg^r	This work
pMD2E1	Derivative of pMhsp60 carrying Mtb *moaD2* and Mtb *moaE1* expressed as an operon under control of the *hsp60* promoter; Hyg^r	This work
pMD2E2	Derivative of pMhsp60 carrying Mtb *moaD2* and Mtb *moaE2* expressed as an operon under control of the *hsp60*	This work

	promoter; Hyg^r	
pMBRD1E2	Derivative of pMhsp60 carrying Mtb *moaD1,* Mtb *moaE2* and *moeBR* expressed as an operon under the control of the *hsp60* promoter; Hyg^r	This work
pFLAGEM	*E. coli-Mycobacterium* episomal shuttle vector carrying the 3× FLAG epitope sequence and the Tet-operator; Hyg^r	This work
pMoaXFL	Derivative of pFLAGEM carrying *moaX* with the 3× FLAG sequence on the C-terminus under the control of the Tet-operator	This work
pMoaXFLG81A	Derivative of pFLAGEM carrying *moaX* with a g→c point mutation at position 242, resulting in a Gly81→Ala change, and the 3× FLAG-tag sequence on the C-terminus under the control of the Tet-operator	This work
pMoaXFLG82A	Derivative of pFLAGEM carrying *moaX* with a g→c point mutation at position 245, resulting in a Gly82→Ala change, and the 3× FLAG-tag sequence on the C-terminus under the control of the Tet-operator	This work

Table S3: Primers used for construction of plasmids

Gene cloned	Primer name	Primer sequence (5'-3') *
Mtb *moaD1*	mD1F	GGCGCTGCAGAATGATTAAAGTGAATGTTCTTTACTTC (*Pst*I)
	mD1R	CGAAGCTTTCAGCCTCCGGCTACCTG (*Hind*III)
Mtb *moaD2*	mD2F	GGCGCTGCAGAGTGACGCAGGTGTCCGA (*Pst*I)
	mD2R	CGAAGCTTTTAGCCGCCGGCGAAAGG (*Hind*III)
moeBR	moeBRF	TTAATTAAATGACTGAAGCCTTGATCCCGG (*Pac*I)
	moeBRR	TTAATTAACTAGTACACCAACAAGGAGGAG (*Pac*I)
moaX	moaXF	GCCGTGTACAGATGATTACTGTCAATGTGCTC (*BsrGI*)
	moaXR	CCGCGTACGCCTGGCCGATTGGCCACCCACTC (*BsiWI*)
	moaXga1R	GACATCGGAGCCC**G**CGGCAACCTGC[a]
	moaXga2R	GACATCGGAG**G**CCCCGGCAACCTGC[a]

*Underlined sequences represent restriction sites with the restriction enzymes used shown in parenthesis. [a]Bold text represents a point mutation.

Table S4: Primers used for q(RT)PCR analysis.

Gene	Primer name	Primer sequence (5’ → 3’)	*Position
sigA	SSF	GGGCGTGATGTCCATCTCCT	367-488
	SSR	GTATCCCGGTGCATGGTC	
Msm *moaD2*	RTSMEGDF	GAATCGAGACCGAATCGTTG	42 -156
	RTSMEGDR	AGGTATGAGCAGCGCTTGAG	
Msm *moaE2*	SMRTEF2	CTACCGAGGACCCGATCT	28-209
	SMRTER	ATCTCCTCGGCGACCTCT	
Mtb *moaD1*	RTTbD1F	TCTTTACTTCGGTGCCGTTC	18-138
	RTTbD1R	AAGGCGAGGGTATTTTTGCT	
Mtb *moaD2*	RTTbD2F	GGTGACTGTCCGCTACTTCG	36-143
	RTTbD2R	GACAGGCCGTCTATCAGCTC	
Mtb *moaE1*	RTE1Fa	GATGTCACGCGGTTGTTCTA	154-391
	RTE1Ra	AAATCGGCACTTCCTGCTTA	
Mtb *moaE2*	RTE2Fa	GCGGAGGTAGCTGAAGAGTC	199-425
	RTE2Rb	TAAACCGAACCCACCCATT	
moaX	RTTbXF	TCGCTCACGAGAAGATCTCA	53-134
	RTTbXR	AGTGGCGGATAGTCGATTTG	
moeBR	BRRTF	ACTTCCTCTTGGTCGACGTG	899-1025
	BRRTR	TCCTTGTCCAGCGGTAGC	

* Position within the gene where primers hybridize

Supplementary Figures

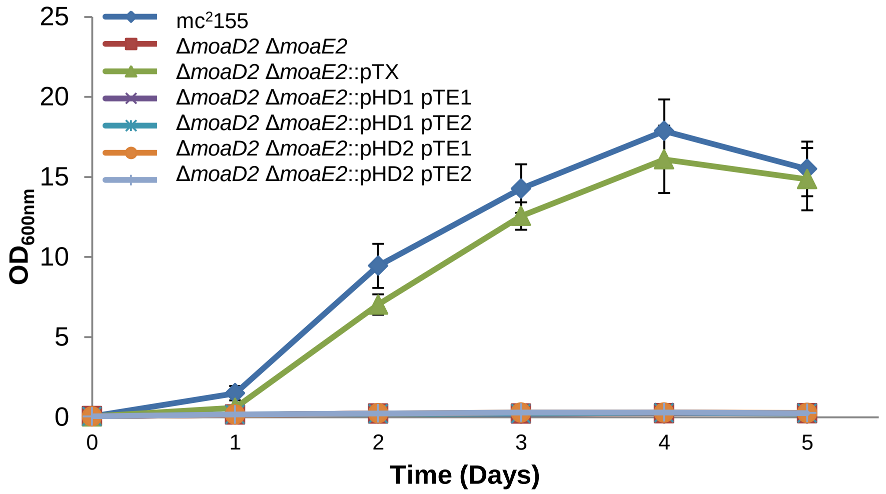

Figure S1. Growth curve of *M. smegmatis* ΔmoaD2ΔmoaE2 complemented with different combinations of Mtb *moaD1*, Mtb *moaD2*, Mtb *moaE1* and Mtb *moaE2* carried on integrating vectors. Growth curves were carried out in MPLN, supplemented with 10 mM, sodium nitrate with optical density readings taken daily for 5 days. The data are an average of at least three independent experiments.

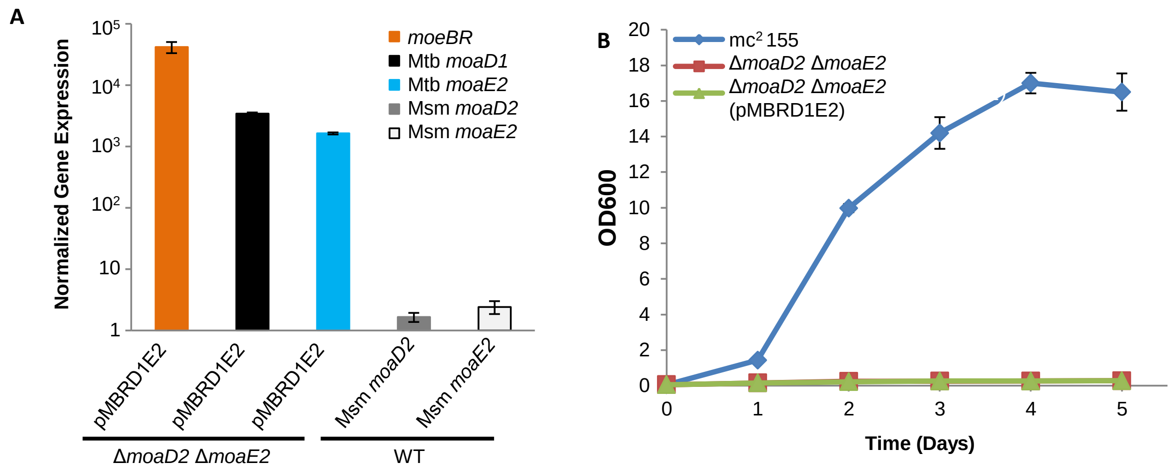

Figure S2. MoeBR does not confer functionality with MoaD1 homologues. (A) Gene expression analysis of *M. smegmatis* double mutant strain complemented with Mtb *moaD1,* Mtb *moaE2* and *moeBR*. The expression of each gene was normalized against *sigA* expression. Data were generated from three independent experiments. **(B)** Nitrate assimilation in *M. smegmatis* strains. Growth curves were carried out in MPLN, supplemented with 10 mM, sodium nitrate. The growth curve is an average of four independent experiments with standard errors.

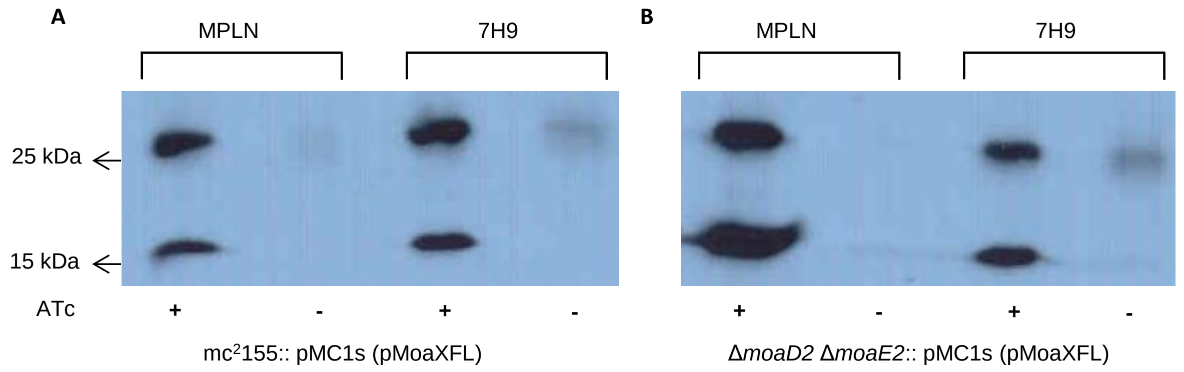

Figure S3. Cleavage of MoaX under different growth conditions. Strains carrying pMoaXFL were transformed with pMC1s, which carries the TetR repressor. In this case, addition of anhydrotetracycline induces expression of *moaXFL*. Cleavage was tested in standard 7H9 broth and in MPLN media **(A)** MoaX cleavage in mc^2155 **(B)** MoaX cleavage in Δ*moaD2* Δ*moaE2*:: pMC1s (pMoaXFL).

References

1. Snapper SB, Melton RE, Mustafa S, Kieser T, Jacobs WR, Jr.: **Isolation and characterization of efficient plasmid transformation mutants of *Mycobacterium smegmatis***. *Mol Microbiol* 1990, **4**(11):1911-1919.
2. Williams MJ, Kana BD, Mizrahi V: **Functional analysis of molybdopterin biosynthesis in mycobacteria identifies a fused molybdopterin synthase in *Mycobacterium tuberculosis***. *J Bacteriol* 2011, **193**(1):98-106.
3. Sassetti CM, Boyd DH, Rubin EJ: **Genes required for mycobacterial growth defined by high density mutagenesis**. *Mol Microbiol* 2003, **48**(1):77-84.
4. O'Gaora P, Bernini, S., Hayward, C., Filley, E., Rook, G, Young, G. B and Thole, J: **Mycobacteria as immunogens. Development of expression vectors for use in multiple species.** . *Med Princ Pract* 1997, **6**:91-96.
5. Ehrt S, Guo XV, Hickey CM, Ryou M, Monteleone M, Riley LW, Schnappinger D: **Controlling gene expression in mycobacteria with anhydrotetracycline and Tet repressor**. *Nucleic Acids Res* 2005, **33**(2):e21.
